# Editorial: Platelets and Immune Responses During Thromboinflammation

**DOI:** 10.3389/fimmu.2020.01079

**Published:** 2020-05-28

**Authors:** Mirta Schattner, Craig N. Jenne, Soledad Negrotto, Benoit Ho-Tin-Noe

**Affiliations:** ^1^Laboratory of Experimental Thrombosis, Institute of Experimental Medicine-CONICET-National Academy of Medicine, Buenos Aires, Argentina; ^2^Department of Microbiology, Immunology, and Infectious Diseases, University of Calgary, Calgary, AB, Canada; ^3^Université de Paris, LVTS, Inserm U1148, Paris, France

**Keywords:** platelets, thromboinflammation, neutrophils, endothelial cells, inflammation, sepsis, cancer

The word thromboinflammation appeared in 2004 to describe the interactions and cooperation between platelets and neutrophils in the context of arterial in-stent restenosis (1). Almost two decades later, multiple sources of evidence clearly show that the interplay between thrombosis and inflammation involves several pathways and occurs in various pathophysiological situations such as sepsis, disseminated intravascular coagulation (DIC), stroke, cancer, stress and rheumatoid arthritis, among others. Thromboinflammation is driven by mutual interactions and reciprocal activation between endothelial cells, subendothelium, leukocytes, platelets, and the humoral innate immune system, involving the complement, coagulation, and fibrinolytic signaling cascades.

In many respects, this linkage between inflammation and thrombosis is not entirely surprising. Some of the earliest examples of innate immunity center on the use of “clotting” or “coagulation” to respond to infectious agents and tissue damage. In some invertebrates, this “clotting” response occurs in the hemolymph and is facilitated by hemocytes, the evolutionary ancestor to the vertebrate platelet (2). In these systems, exposure of hemocytes, or hemolymph to bacteria results in rapid coagulation of the hemolymph, trapping the pathogen, and limiting its dissemination (3). This early basic response has evolved to become more specialized, creating distinct roles of hemostasis, inflammation, and immunity; however, the evolutionary overlap in these processes remain and continue to play a role in a wide variety of pathophysiological conditions and human disease.

In this Special Research Topic issue on the recent advances in Thromboinflammation, we compiled 15 reviews and one original article which provide comprehensive basic, and clinical insights on the current view of thromboinflammation. These articles also address the development and use of novel therapeutics in both experimental settings as well as in clinical trials, moving our understanding of the topic forward within the context of treating human disease.

Long before the term thromboinflammation was coined, interactions between neutrophils and platelets had been suspected to play a pathophysiological role in both myocardial and cerebral ischemia-reperfusion injury (4–7). Schanze et al. and Stegner et al. present early and recent findings on the crucial contribution of platelets to the course of ischemia-reperfusion injury and discuss how our current understanding of the underlying molecular mechanisms could translate into the clinics. A particular focus on the role of the von Willebrand factor (VWF)/glycoprotein Ibα (GPIbα) axis in the thromboinflammatory response during ischemic stroke is given by Denorme et al.. This work explores the “thrombo-inflammatory” environment surrounding VWF-platelet interactions within the context of cardiovascular diseases and how these players may serve as both important clinical markers of disease and also key therapeutic targets for the treatment of stroke patients.

Recent years have seen a growing interest for the impact of the gut microbiome on the development of diseases with an immune and/or thrombotic component, such as cardiovascular diseases. In this context, Bayer et al., highlight the fact that antibiotics-based protocols commonly used in this research field can significantly alter host physiology, notably by impacting the cardiovascular system and the actors of the thromboinflammatory cascade. The authors emphasize the importance of instead considering the use of germ-free mouse models, which remain the state-of-the-art approach for studying host-microbe and microbe-microbe interactions.

Beyond cardiovascular health, inflammation is often associated with the development and progression of cancer. Emerging in this field is the view that considers malignant tumors as wounds that do not heal. In this scenario, platelets are intimately involved in the vicious circle of activating cancer cells which, in turn, activate platelets. This mutual cell activation can ultimately end in the well-recognized cancer-associated thrombosis or can further fuel the inflammatory microenvironment which favors tumor growth and metastasis. Palacios-Acedo et al. give a detailed description of the molecules and mechanisms in these events, while Marin Oyarzún and Heller summarize current knowledge about the role of platelets in the thrombotic complications of chronic myeloproliferative neoplasms.

The crossroad of inflammation and thrombosis is very well-exemplified during envenomation. Several characterized proteins in particular from viper snake venoms can activate the innate immune and/or hemostasis systems. Teixeira et al. offer and extended review of these molecules and its mechanisms of action in this elaborate interplay between predator venom and host hematology.

Platelets and coagulation are often centrally involved in the host response to infectious disease as well. Manifestations of dysregulated coagulation, such as DIC, are frequently associated with the systemic immune response, such as in the case of sepsis, and are almost invariantly linked to worse patient outcomes. In this issue, reviews by Kerrigan et al. and by McDonald and Dunbar explore how this dysregulated response is initiated in the septic patient and how its manifestation impacts both the infection itself (protective) and vascular health (pathogenic). In a complementary review, Assinger et al. give a timely update on experimental models of sepsis, highlighting both the interest and limitations of animal models to study platelet-related functions in sepsis.

Additional contributions address the role of the platelet as an immune sentinel, detecting and initiating the response to infection. Guo and Rondina address the broad ability of platelets to respond to Pathogen Associated Molecular Patterns (PAMPs), complement and antibodies and how activated platelets in turn drive and modulate the host immune response. The authors also address how this host immune response is intimately intertwined with both the hemostatic and thrombotic pathways, directly impacting patient outcomes in infectious disease. Ramirez et al. build on this theme, shedding light on the direct interplay between platelets and neutrophils within the context of chronic inflammation. Importantly, this work also addresses both platelet production within the bone marrow of patients with chronic disease and the role of platelet-derived microparticles within chronic inflammation. The role of the platelet as an innate immune cell is further discussed in the review by Eriksson et al., in which the interactions between activated platelets and the complement system, as well as their clinical implications, are thoroughly presented. Cognasse et al. broaden our perspective of the immunomodulatory properties of platelets by presenting how platelets can themselves release non-infectious danger signals during storage of platelet concentrates, thus contributing to adverse immune reactions to platelet transfusion.

Additionally, a review by Mezger et al. gives an overview of several non-hemostatic functions of platelets, including the regulation of angiogenesis, tissue remodeling, and other processes contributing to the progression of various thromboinflammatory conditions like cancers and cardiovascular diseases.

Finally, in an original article and by using a pre-established mouse model of oral acute Chagas disease, Antunes et al. demonstrate for the first time that *Trypanosoma cruzi* infection leads to a decrease in platelet count, increased bleeding and coagulation time, host responses that correspond to the peak of parasitemia. Importantly, circulating IL-6 levels seem to be involved in these hematological changes during oral Trypanosoma cruzi infection. These data may help elucidate the mechanism of oral acute Chagas disease pathogenesis and provide additional insight on the interaction between inflammation and coagulation in the context of infectious diseases.

Overall, this collection of manuscripts expands our understanding of the non-hemostatic roles of platelets, highlighting the central role thrombocytes play in linking coagulation, inflammation, and immunity by participating in a spectrum of immunothrombotic responses and directly impacting host responses and disease outcomes.

## Author Contributions

All authors listed have made a substantial, direct and intellectual contribution to the work, and approved it for publication.

## Conflict of Interest

The authors declare that the research was conducted in the absence of any commercial or financial relationships that could be construed as a potential conflict of interest.
